# Feasibility and Postoperative Outcome After Duet Procedure for Reversible Multifocality in Eyes with Co-Pathologies

**DOI:** 10.3390/jcm14155583

**Published:** 2025-08-07

**Authors:** Barbara S. Brunner, Martin Dirisamer, Nikolaus Luft, Stefan Kassumeh, Siegfried G. Priglinger

**Affiliations:** 1 Department of Ophthalmology, LMU University Hospital, LMU Munich, 80336 Munich, Germany; barbara.brunner@med.uni-muenchen.de (B.S.B.); martin.dirisamer@med.uni-muenchen.de (M.D.); nikolaus.luft@med.uni-muenchen.de (N.L.); stefan.kassumeh@med.uni-muenchen.de (S.K.); 2 Auge Laser Chirurgie, Weissenwolffstrasse 13, 4021 Linz, Austria

**Keywords:** premium IOLs, multifocal IOLs, cataract surgery, duet implantation, accommodative lenses, refractive lens exchange, polypseudophakia, spectacle independence

## Abstract

**Objectives**: To evaluate the safety and efficacy of the simultaneous implantation of a monofocal capsular bag-fixated and a trifocal supplementary sulcus-fixated intraocular lens (duet procedure) in eyes with co-existing pathologies undergoing cataract or refractive lens surgery. **Methods**: In total, 80 eyes of 40 consecutive patients, who underwent refractive lens exchange or cataract surgery and received the duet procedure due to minor co-pathologies, were included in this retrospective case series. Preoperative assessment comprised slit-lamp biomicroscopy, optical biometry, posterior-segment optical coherence tomography, corneal endothelial specular microscopy, corneal tomography, manifest refraction and distance and near visual acuity testing. Three months postoperatively, uncorrected distance (UDVA) and uncorrected near visual acuity (UNVA) were recorded. **Results**: The preoperative manifest refractive spherical equivalent (MRSE) was −0.31 ± 4.29 diopters (D), with a mean refractive astigmatism of −0.80 ± 0.60 D. At three months postoperatively, monocular UDVA and binocular UNVA significantly improved from 0.52 ± 0.42 logMAR and 0.32 ± 0.27 logMAR to 0.05 ± 0.09 logMAR and −0.03 ± 0.10 logMAR, respectively (both *p* < 0.0001). **Conclusions**: Reversible multifocality provided by the duet procedure appears to be a feasible option in eyes with mild co-existing pathologies, as it yields satisfactory visual and refractive outcomes with high safety.

## 1. Introduction

Advancements in measurement technology and novel intraocular lens (IOL) models allow for more precise refractive corrections and individualized care in cataract and refractive lens surgery. Globally, the desire for spectacle independence is soaring. Nevertheless, only approximately 5% of all cataract patients receive multifocal intraocular lenses (IOLs) [1]. While acceptable visual acuity for distance, intermediate and near vision can be accomplished, a major concern is unwanted visual phenomena, such as halos or glare as well as reduced contrast sensitivity [2]. In rare cases, those side effects can be intolerable and may necessitate an IOL exchange, which is problematic due to its complications, namely, capsular rupture, vitreous loss and zonular dehiscence [3]. Therefore, the duet implantation of a capsular bag-mounted monofocal IOL and a sulcus-fixated multifocal IOL offers an easier reversible approach [4]. While the concept of implanting two IOLs in one eye (polypseudophakia) was first described in 1993 to correct higher ametropia when the IOL power of a single IOL was insufficient [5], the technique has since gained popularity, e.g., in the form of an additional sulcus-fixated IOL to correct for postoperative refractive errors [6] or to meet a subsequent desire for spectacle independence [1].

In addition, some ocular co-pathologies themselves lead to light aberrations, reduced contrast sensitivity, leading to diminished overall visual quality. For example, patients with Fuchs endothelial corneal dystrophy (FECD) and a modified Krachmer grade of ≥3 suffer from decreased visual function [7]. Further, patients with epiretinal membranes show a significantly reduced contrast sensitivity and visual acuity with increasing central retinal thickness [8]. Depending on the state and the course of the disease, especially the development and severity of visual field defects, glaucoma can also entail unsatisfactory outcomes with a trifocal IOL [9]. As of today, the aforementioned pathologies are commonly regarded as contraindications for the use of trifocal intraocular lenses [10].

Currently, the duet procedure is a particularly preferred option in young, occupational patients with high visual demands, who might not tolerate the side effects of multifocal optics. Not only in cases of intolerable optic phenomena and/or contrast sensitivity loss but also with later development of ocular co-pathologies incompatible with a trifocal optic (e.g., age-related macular degeneration), patients can benefit from the reversible nature of the duet procedure [11,12]. Complication rates of explanting a sulcus-fixated IOL are significantly lower compared to an in-the-bag IOL exchange [1]. At the same time, visual and refractive outcomes as well as complication rates of the duet procedure are non-inferior to the implantation of a single multifocal capsular bag-mounted multifocal IOL [13]. Furthermore, in cases of a refractive surprise, the duet procedure enables a less complicated postoperative exchange of the sulcus-fixated IOL according to the manifest refraction [14,15].

While there is sufficient proof of feasibility and safety of the duet procedure in cataract or refractive lens surgery in otherwise healthy eyes [11], data in eyes with pre-existing co-pathologies is scarce. To this end, the following study investigated the safety and efficacy of the duet procedure in a heterogeneous consecutive sample of patients with non-lens-related ocular co-pathologies seeking spectacle independence. Therefore, visual and refractive outcomes as well as postoperative complications following the duet procedure were addressed.

## 2. Materials and Methods

The present study is a retrospective consecutive case series. Ethical approval was obtained from the local institutional review board of the Ludwig Maximilian University (LMU) before data collection and analysis (approval number: 24-0882). This study complied with the guidelines set forth in the Declaration of Helsinki.

### 2.1. Inclusion and Exclusion Criteria

All patients who underwent cataract surgery or refractive lens exchange with the implantation of a capsular bag-fixated monofocal IOL and a supplementary trifocal sulcus-fixated IOL simultaneously between February 2022 and April 2024 were screened for inclusion in this study. Patients with minor co-pathologies—such as mild Fuchs endothelial corneal dystrophy (maximum Krachmer G4), non-tractive epiretinal membranes (maximum stage 1), mild non-proliferative diabetic retinopathy, macular drusen due to early AMD (exclusion criteria: intermediate ARD or severe) or retinal pigment epithelial alterations due to preceding central serous chorioretinopathy—were included.

Exclusion criteria were the presence of pigment dispersion syndrome, perimetric glaucoma (with manifest visual field defects), traumatic or PEX-induced zonulolysis and posterior synechiae. Patients with amblyopia and a documented visual acuity of less than 20/32 (logMAR equivalent = 0.20) were also excluded.

### 2.2. Perioperative Assessment

All study participants were examined in detail both preoperatively and 12 weeks postoperatively. Preoperatively, the uncorrected distance (UDVA) and near visual acuity (UNVA), as well as the best-corrected distance visual acuity (BCDVA) after manifesting subjective refraction, were assessed. Furthermore, slit lamp biomicroscopy of the anterior segment as well as a dilated fundus exam were performed. Pneumotonometry was performed with the Tonoref II (Nidek Co. Ltd., Gamagōri, Japan) device. A morphological image of the retina using optical coherence tomography (OCT) was acquired (Revo FC, Optopol Technology Sp., Zawiercie, Poland). Biometric and topographic data were obtained with optical biometry (IOLMaster 500, Carl Zeiss Meditec AG, Jena, Germany) and anterior segment optical coherence tomography system with integrated Placido disc technology (MS-39, Costruzione Strumenti Oftalmici, Firenze, Italy). Endothelial cell morphology and density were assessed with specular microscopy (NIDEK CEM-530). Postoperatively, pneumotonometry, manifest refraction, UDVA, UNVA, slit lamp biomicroscopy of the anterior segment as well as central retinal OCT were assessed.

### 2.3. Surgical Procedure and Intraocular Lens Selection

All cataract and refractive lens surgeries were performed by a single highly experienced surgeon (S.G.P.) at a single tertiary care centre (Department of Ophthalmology, LMU Hospital). Surgeries were performed under topical anesthesia. In brief, a 2.5 mm clear corneal incision (CCI) was performed at the temporal corneal limbus. After instillation of lidocaine, epinephrine and a dispersive ophthalmic viscosurgical device (OVD) two paracentheses were created, each two clock hours away from the CCI. A curvilinear capsulorhexis was performed using a cystotome. After phacoemulsification using the phaco-chop technique, residual lens cortex was removed via bimanual irrigation and aspiration. After filling the capsular bag with dispersive OVD, a monofocal IOL was implanted. In the case of a toric monofocal IOL, axis marking was performed using a digital marking system (Callisto^®^; Carl Zeiss Meditec AG, Jena, Germany). After removal of OVD from behind the IOL with irrigation and aspiration, the ciliary sulcus was filled with dispersive OVD. Subsequently, the trifocal sulcus-fixated IOL was implanted (Sulcoflex Trifocal 703F; Rayner, Worthing, UK). Again, using irrigation and aspiration, the OVD was removed thoroughly. To prevent postoperative iris capture, acetylcholine (1%, 0.5 mL) was instilled to constrict the pupil. Thereafter, 1 mg of cefuroxime in 0.1 mL was also injected into the anterior chamber before wound closure with balanced salt solution hydration of the incisions. Eventually, dexamethasone 0.1% and tobramycin 0.3% ointment (Tobradex; Novartis AG, Basel, Switzerland) was administered topically, and the eye was covered with a sterile patch for 12 h.

Target refraction for the primary capsular bag-fixated IOL was set to plano in 38 of 40 patients. In patients with low myopic refractive error prior to surgery, the target refraction of the primary capsular bag-fixated IOL can be set to −2.50 D to maintain optimal unaided near vision in the event of supplementary IOL explantation (multifocal myopic duet implantation; MMDI) [4]. In our study, two patients opted for this approach. The refractive power of the sulcus-fixated IOL was adapted accordingly to achieve a plano outcome.

### 2.4. Data Management and Statistical Analysis

All statistical analysis was performed using Prism 10 (Version 10.4.2, GraphPad Software, San Diego, CA, USA). All numerical data are expressed as mean ± standard deviation (SD) and range. Standard refractive outcomes were graphically computed using the mEYEstro software by Gauvin and Wallerstein [16]. The Shapiro–Wilk test was used to test for normal distribution. To compare visual acuity outcomes, the paired t test was used. A *p*-value less than 0.05 was considered to be statistically significant. To reduce selection bias, patients were included by five independent refractive surgeons.

## 3. Results

### 3.1. Patient Demographics and Baseline Parameters

A total of 80 eyes of 40 patients were included in this study. Subjects’ baseline characteristics and biometric features are summarized in Table 1.

### 3.2. Co-Pathologies

The leading co-pathology was dry eye disease (23%; 9 of 40 patients), followed by a non-tractive epiretinal membrane (18%; 7 of 40 patients) and macular drusen as well as pigment epithelial alterations (18%; 7 of 40 patients). Further, five patients (13%) suffered from mild Fuchs’ endothelial corneal dystrophy (FECD), three from mild unilateral amblyopia (8%; visual acuity ≥ 20/32) and three (8%) from myopic maculopathy. In addition, three patients (8%) showed optic nerve head alterations (optic drusen; suspicious excavation; glaucoma suspect). One patient presented with mild epithelial basement membrane dystrophy (3%), one with mild diabetic retinopathy (3%) and one with a history of strabismus surgery (3%).

Of the 40 patients undergoing surgery, 12 (30%) suffered from cataract, while the other 28 (70%) underwent refractive lens exchange.

### 3.3. Intraocular Lens Models

In total, four different capsular bag-fixated IOLs were used. In 27 (34%) of the eyes, the Avansee CP2.2R (Kowa Company Ltd., Tokyo, Japan) was implanted, and, in 22 (25%) of the eyes, the CT Asphina 409MP (Carl Zeiss Meditec AG, Jena, Germany) was implanted. In 22 (28%) of the eyes with significant astigmatism, the AT Torbi 709MP (Carl Zeiss Meditec AG, Jena, Germany) was implanted, while 11 (13%) eyes received an Ankoris toric (BVI Medical, Waltham, MA, USA). In total, astigmatism correction via the implantation of a toric intraocular lens was performed in 33 eyes (41%).

### 3.4. Visual Acuity and Refractive Outcomes

**Figure 1:**
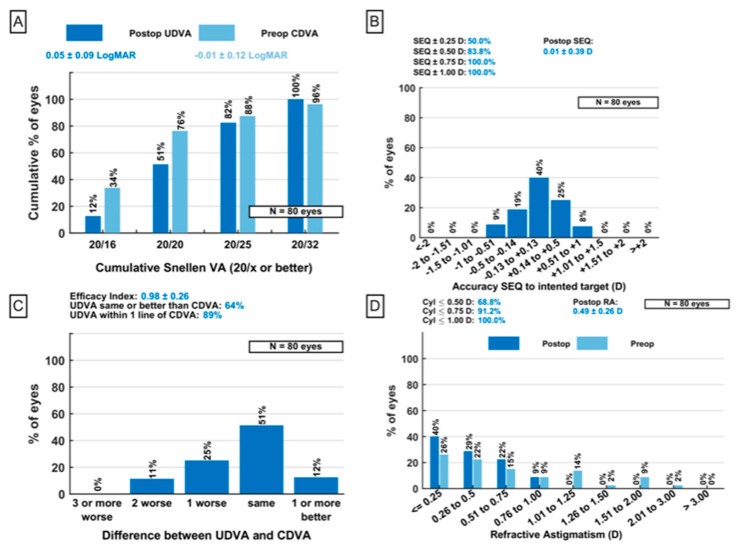
**Refractive and visual acuity outcomes three months postoperatively:** (**A**) Cumulative Snellen uncorrected and corrected distance visual acuity. (**B**) Postoperative residual spherical equivalent. (**C**) Efficacy index: Difference in Snellen visual acuity lines when postoperative UDVA and preoperative CDVA are compared. (**D**) Comparison of refractive cylinder pre- and postoperatively. UDVA = uncorrected distance visual acuity; CDVA = corrected distance visual acuity; VA = visual acuity; and D = diopters.

The achieved MRSE was 0.01 ± 0.39 D (range: −0.75–+0.75 D) at three months postoperatively. Detailed visual acuity and refractive outcomes are illustrated in Figure 1. In total, all eyes reached a postoperative UDVA of 20/32 or better (Figure 1A). None of the included eyes lost more than two lines of UDVA postoperatively compared to CDVA preoperatively (Figure 1C). Postoperative refractive astigmatism was ≤ 1.00 D and MRSE was ≤ 0.75 D for all operated eyes (Figure 1B,C). Mean preoperative monocular UDVA was 0.52 ± 0.42 logMAR (range: 1.40–0.10 logMAR) and increased significantly to 0.05 ± 0.09 logMAR (range: 0.20–0.20 logMAR) three months postoperatively (*p* < 0.0001). The binocular uncorrected near visual acuity (UNVA) significantly increased from 0.32 ± 0.27 logMAR (range: 0.70–0.10 logMAR) to −0.03 ± 0.10 logMAR (range: 0.20–0.10 logMAR) three months after the surgery (*p* < 0.0001).

### 3.5. Complications and Use of the Reversibility

Due to a myopic outcome on the left eye, one (3%) patient underwent monocular exchange of the supplementary IOL. Due to postoperative rotation of the capsular bag-mounted toric IOL in one (3%) other patient, an IOL rotation of the intracapsular IOL was performed 7 weeks postoperatively. One (3%) patient suffered from postoperative cystoid macular edema four weeks after surgery, which completely resolved after parabulbar injection of 40 mg triamcinolone.

## 4. Discussion

To our knowledge, this is the largest cohort evaluating the duet procedure in eyes with co-existing ocular pathologies. Our results endorse the safety and efficacy of the multifocal duet procedure in eyes with minor co-pathologies undergoing refractive lens exchange or cataract surgery. The achieved visual acuity outcomes are in line with studies on capsular bag-fixated trifocal IOLs in eyes with co-pathologies. We observed a postoperative UDVA of 0.05 ± 0.09 logMAR (corresponding to 20/20 Snellen) and a binocular UNVA of −0.03 ± 0.10 logMAR (20/20 Snellen). Zhu et al. reported a UDVA of 0.04 ± 0.07 logMAR (20/20 Snellen) and a UNVA of 0.12 ± 0.10 logMAR (20/25 Snellen) following the implantation of a trifocal capsular bag-fixated IOL (AT LISA tri 839MP, Carl Zeiss AG, Oberkochen, Germany) in high myopic eyes (axial length > 26.0 mm) [17]. Kim et al. implanted capsular bag-fixated trifocal IOLs (Panoptix TFNT, Alcon Laboratories Inc., Fort Worth, TX, USA) in eyes with a non-foveal involving epiretinal membrane (ERM). Of the 92 eyes included in the ERM group of this study, 95% reached a UDVA and 98% a UNVA of 0.10 logMAR (20/25 Snellen) or better [18]. When compared to their control group without a non-foveal ERM, the authors observed no significant difference in visual acuity results between the groups [18]. Our visual acuity results are also non-inferior to those reported by Blau-Most and co-authors [19]. Their main outcome was the visual acuity after implantation of a capsular bag-fixated trifocal intraocular lens in eyes with mild to moderate FECD without significant corneal edema (confluent guttae up to an area of 5 mm). In total, 10 eyes with FECD received a trifocal IOL (either Finevision Micro F or Pod FT, BVI Medical; or SN6AD1 or SND1T4, Alcon Laboratories Inc.), whereas 30 healthy eyes served as a control group. The UDVA accounted for 0.06 ± 0.11 logMAR (20/25 Snellen) and 0.03 ± 0.07 logMAR (20/20 Snellen) for the UNVA group, respectively, with no statistical difference from the control group [19]. While those previously mentioned studies refer to a capsular bag-fixated trifocal intraocular lens, Harrisberg et al. proved that the primary duet IOL procedure is equally effective and safe in correcting distance and near vision when compared with a single multifocal IOL in the capsular bag [13].

While the implantation of two capsular bag-fixated intraocular lenses, sometimes also referred to as “polypseudophakia”, has a long history, it was primarily used in high ametropic patients, in which one single IOL could not provide sufficient dioptric power [20]. With the emergence of supplementary sulcus-fixated IOL, Prof. Michael Amon was the first to coin the term “duet procedure” in 2009, referring to the simultaneous implantation of a capsular bag-fixated IOL and a supplementary sulcus-fixated IOL in one surgery [21]. Since then, supplementary sulcus-fixated IOLs have been proven feasible not only for the correction of postoperative residual refractive errors [22] but also to improve monofocally implanted patients’ spectacle independence [1,23]. More recently, Baur et al. endorsed the concept of reversible multifocality via the primary duet procedure, emphasizing its advantages in cases requiring explantation or exchange of the trifocal IOL. In their case report, a 49-year-old woman suffering from hypermetropia, astigmatism, and presbyopia was treated with the duet procedure to obtain spectacle independence. Three months postoperatively, an evident myopic outcome of nearly −1.00 D on both eyes led to unsatisfactory uncorrected distance and near visual acuity. Six months after the surgery, both supplementary intraocular lenses were exchanged, finally leading to a satisfactory visual acuity and near emmetropic refraction [14]. In our present study, we took advantage of the benefits of the reversible nature since we had to monocularly exchange the supplemental IOL in one patient due to a myopic outcome.

In a second case report, Khoramnia et al. proved feasibility of the duet procedure in a young patient with presenile cataract due to hereditary hyperferritinemia-cataract syndrome. The reason for this approach was again the reversible nature since this patient might develop other ophthalmic conditions later in life that could not be anticipated at the time of surgery [12]. In the first retrospective duet implantation case series by the same group [11], 25 patients undergoing refractive lens exchange or cataract surgery were included. The decision to perform the duet procedure was based on the following criteria: (1) young age; (2) high refractive error; (3) subtle morphologic changes (e.g., RPE alterations); (4) doubtful tolerance for a trifocal optic; and (5) history of strabism/mild amblyopia. The authors provided excellent visual acuity and refractive results and concluded that the procedure is feasible in the above-mentioned cases and still has the advantage of an exit strategy in cases with a future loss of function or side effects associated with the optics. While our study confirms their findings, it adds to the body of evidence on the duet procedure in eyes with minor corneal or retinal co-pathologies, such as FECD, dry eye disease, extrafoveal ERM, mild diabetic retinopathy or optic disc drusen.

Our study faces minor limitations that should be addressed: (1) This study is mainly limited by its retrospective nature. (2) The study cohort was heterogeneous, including patients undergoing either clear lens exchange or cataract surgery, and presenting with various co-pathologies. (3) Multiple models of capsular bag-fixated IOLs were used.

In conclusion, reversible multifocality achieved by the duet procedure may be considered in eyes with mild co-pathologies, including non-tractive epiretinal membranes, mild FECD, mild amblyopia or macular drusen. In the case of long-term disease progression and associated declines in contrast sensitivity or visual acuity that render multifocal optics intolerable, the supplementary trifocal IOL can be removed with relative ease.

## Figures and Tables

**Table 1:** Patient demographics and baseline parameters preoperatively.

Parameter	Mean ± SD [Minimum; Maximum]
Age [years]	60 ± 6 [43; 72]
Gender [male/female]	21:19
Spherical Equivalent Preoperatively [D]	−0.31 ± 4.29 [−13.50; 5.88]
Refractive Cylinder Preoperatively [D]	−0.80 ± 0.60 [−2.75; 0.00]
Axial Length [mm]	23.72 ± 1.57 [21.50; 28.86]
Anterior Chamber Depth (internal) [mm]	2.77 ± 0.41 [2.02; 3.62]

## Data Availability

Dataset available upon request from the authors.
